# Improvements in health-related quality of life and function in middle-aged women with chronic diseases of lifestyle after participating in a non-pharmacological intervention programme: A pragmatic randomised controlled trial

**DOI:** 10.4102/ajod.v8i0.428

**Published:** 2019-02-25

**Authors:** Roline Y. Barnes, Jennifer Jelsma, Romy Parker

**Affiliations:** 1 Department of Physiotherapy, University of the Free State, South Africa; 2 Division of Physiotherapy, Department of Health and Rehabilitation Sciences, University of Cape Town, South Africa; 3 Department of Anaesthesia and Perioperative Medicine, University of Cape Town, South Africa

## Abstract

**Background:**

Musculoskeletal diseases consume a large amount of health and social resources and are a major cause of disability in both low- and high-income countries. In addition, patients frequently present with co-morbid chronic diseases of lifestyle. The area of musculoskeletal disease is restricted by a lack of epidemiological knowledge, particularly in low- and middle-income countries.

**Objectives:**

This pragmatic randomised controlled trial assessed the benefits of a 6-week physiotherapy intervention for middle-aged women with musculoskeletal conditions compared to usual care.

**Method:**

A weekly 2-h educational programme utilising a workbook, discussion group and exercise class was presented for the intervention group, while the control group received usual care. The primary outcome was health-related quality of life. Parametric and non-parametric data were used to determine the equivalence between the groups.

**Results:**

Twenty-two participants were randomised to the intervention and 20 to the control group. The control group demonstrated no within-group improvement in health-related quality of life items, compared to significant improvements in two items in the intervention group. The change in median utility score within the intervention group was twice as large as the change in the control group. With regard to self-efficacy, the intervention group demonstrated significant within-group changes in perceived management of fatigue and discomfort.

**Conclusion:**

The positive impact of the intervention on the participants suggests that the programme should continue at the clinic in question, but should be presented at a more convenient time for participants who work, as recruitment to the study was less than anticipated. Primary health care systems in South Africa urgently need to put structures in place for effective management of the functional impact of chronic diseases of lifestyle and musculoskeletal conditions. It is time for physiotherapists and possibly other health care professionals to participate in the development of appropriate community level interventions to address the functioning and quality of life of individuals living with the diseases.

## Introduction

Musculoskeletal conditions (MSC) consume a large amount of health and social resources and are a major cause of disability in both low- and high-income countries (Brooks 2005; Carmona et al. 2001; Chopra 2009). It is predicted that ageing populations will require more and more relief from chronic pain and disability and that the prevalence of MSC will continue to rise (Connelly, Woolf & Brooks 2006). This is particularly true in South Africa, because of the epidemiological transition that is rapidly taking place. In South Africa, the majority of the population are cared for in primary health care clinics (PHC) that currently provide basic services to low-income communities in urban, peri-urban and rural areas (Lewis, Eskeland & Traa-Valerezo 2004) and are responsible for the ongoing management of chronic diseases of lifestyle. At present, the management of these conditions relies heavily on pharmacological management, which may be expensive and can result in unwanted side effects. A holistic approach to the care of MSC might be more effective and impact not only on the attendant pain and functional limitations of MSC but also on the management of other chronic diseases of lifestyle.

Preventative strategies, for example encouraging moderate physical activity, encouraging weight loss, eating nutritious foods, not using tobacco products and avoiding the consumption of alcohol (Pfleger 2007), can, to some extent, ameliorate the growing burden of disease because of ageing (Brooks 2006). A large percentage of chronic diseases of lifestyle are attributed to sedentary lifestyles, and the prophylactic effects of physical exercise have been described and researched (Connelly et al. 2006). Many of the preventative strategies for both MSC and chronic diseases of lifestyle including hypertension, diabetes mellitus type II and obesity are therefore underpinned by moderate physical exercise and proper nutrition (Lee et al. 2012).

The beneficial effects of exercise interventions, which target behaviour, have been demonstrated in several South African studies. For example, an improvement in health-related quality of life (HRQoL) and health-related behaviours was documented in employees of clothing or textile manufacturing companies in South Africa who participated in a 6-week intervention programme utilising education, exercises, goal setting and pacing (Edries, Jelsma & Maart 2013). Another study on the management of pain in a group of South African women from similar socio-economic and cultural background to those targeted in this study was performed by Parker, Jelsma and Stein (2016). The study concluded that a 6-week peer-led exercise intervention, supported by a workbook, was an efficacious method to treat pain in amaXhosa women living with HIV or AIDS in South Africa (Parker et al. 2016). It was thus hypothesised that the development of an effective exercise and education programme using cognitive behavioural principles (Stajkovic & Luthans 2002), specifically targeting chronic diseases of lifestyle and MSC through lifestyle changes and exercise, could possibly improve the HRQoL of participants. Cognitive behavioural principles are based upon the social cognitive theory, a theory involving a triadic reciprocal causation model in which understanding, behaviour and the environment all dynamically influence each other (Gist & Mitchell 1992). The theory includes motivational and self-regulatory mechanisms and explains the nature of the bidirectional reciprocal influences through five basic human competencies including vicarious learning, symbolising, forethought, self-reflection and self-regulation (Stajkovic & Luthans 2002). As noted, the model has worked well in the South African context, but has not yet been tested within a PHC context with women with MSC who are at risk for chronic diseases of lifestyle.

A non-pharmacological 6-week programme, informed by the results of a systematic review (Barnes 2016) conducted by the first author and incorporating elements of the programmes used in the above two South African studies, was developed. The programme considered adult learning principles, dietary information (determined during the survey), as well as practical considerations and safety aspects in its development. This study aimed to determine the effectiveness of this intervention in middle-aged women presenting with a musculoskeletal disorder, with or without the co-morbidities of hypertension, obesity or diabetes mellitus type II. The objectives were to determine whether the programme would result in a significant difference in HRQoL (primary outcome measure), physical function and self-efficacy.

## Methodology

A pragmatic (Alford 2006), experimental randomised controlled design was undertaken in a PHC clinic in the Free State Province of South Africa. As MSC are more common in middle-aged women (Brooks 2005; Minas et al. 2010; Rollman & Lautenbacher 2001), and the intervention was intended to be contextually and gender relevant, women between the ages of 40 and 64 years who screened positive for MSC, using the COPCORD screening tool (COPCORD), were recruited. The participants were required to have a minimum literacy level equivalent to 4 years of schooling to be able to complete and understand the workbook. Musculoskeletal conditions was defined as experiencing pain, aching, swelling or stiffness in or around joints or back which was not related to an injury or accident. Participants also needed to be able to understand English and or Sesotho, have access to a telephone and be willing to commit to the intervention. Participants with co-morbidities including hypertension, diabetes mellitus type II and or obesity were eligible for inclusion. However, those with other chronic diseases including stroke, depression, cancer, cardiovascular diseases (coronary heart disease) and chronic respiratory diseases were excluded. Those who were assessed as being at risk for exercise as determined by a qualified medical practitioner, and those with diagnosed neurological disorders or confined to a wheelchair, were also excluded.

A survey of MSC was conducted prior to the intervention study (Barnes, Jelsma & Parker 2018). Those participants of the survey who had indicated their willingness and commitment to take part in the intervention were contacted. As it was hypothesised that the body mass index (BMI) of the participants might have an influence on the outcome, eligible women who confirmed their participation in the study telephonically were stratified according to three BMI levels (normal weight: BMI 18.5–24.9; overweight: BMI 25–30; obese: BMI >30) (World Health Organization 2015). Randomisation was carried out for each group separately by the first author, using the Microsoft Excel random function to randomly divide the participants into a usual care group (control group) and an experimental group utilising the workbook. The sample size was based on previous studies in similar samples which utilised the primary outcome measure, the Visual Analogue Scale (VAS) of the EQ-5D instrument in Cape Town (Edries et al. 2013; Jelsma & Ferguson 2004). There is generally a large range of responses to the EQ-5D-3L, as evident by the large standard deviations typically reported. Values include the population norm of 78.55 (standard deviation [SD] 16.57) in Western Australia, 52 (SD = 19) reported by persons with back pain in the UK, and 66.8 (SD = 20.8) and 66.1 (SD 21.3) within samples of people with disabilities living, respectively, in urban and rural areas of South Africa. As it was anticipated that the current sample would be more homogenous, an SD of 15% was chosen for the sample size calculation (Janssen et al. 2013; McCaffrey et al. 2016). An estimated 37 participants were required in each group to detect a difference of 10%, with a SD of 15, a power level of 80% and a *p*-value of 0.05 (Statistica version 7) (StatSoft, Tulsa, Oklahoma, USA).

The first author invited 182 women to participate in the study. The pilot study included 10 women to determine the viability and suitability of the intervention. The rest of the willing women were then included for the implementation of the intervention.

### Instrumentation

The 6-week intervention programme used in the study was developed utilising a workbook that was modified and adapted from previous workbooks successfully used in South African studies (Parker 2013; Parker et al. 2014;). Adult learning principles were applied, and the intervention included physical exercise in group format, health education, facilitation of self-efficacy and self-management, decision-making skills, problem-solving skills and maintaining a balanced lifestyle. Further included was dietary information, as well as safety aspects and practical considerations (Parker 2013). A time period of 6 weeks was used for the programme as this is regarded as the minimum time required to effect a change in the HRQoL of individuals (Parker 2013) while also being a period of time which is not regarded as excessively long by patients (Foster et al. 2007). The frequency of the programme was once a week with a duration of 2 h (1 h education and 1 h supervised exercises) (Edries et al. 2013; Fagard 2001; Greer & Ostwald 2015; Kelley & Kelley 2000; Kruger-Jakins et al. 2016; Oliver & Cronan 2005; Parker 2013).

Standardised questionnaires were used to determine the HRQoL and self-efficacy of the participants. Health-related quality of life measured with the EQ-5D-3L was the primary outcome of the study. The EQ-5D-3L is a single index, generic measurement instrument devised to measure the health of an individual, developed by the EuroQol Group (EuroQol Group 1990). The EQ-5D-3L uses five domains of function to investigate the HRQoL of the individual: mobility, self-care, usual activities (this includes study, work, housework, family or leisure), pain or discomfort, and lastly depression or anxiety. Participants are required to indicate whether they perceive themselves as having no problems, moderate problems or extreme problems with each domain. A vertical VAS is also included in the instrument on which participants indicate their own perceived health state on a scale from 0 to 100, where the end points are labelled ‘worst imaginable health state’ and ‘best imaginable health state’ (Van Reenen & Oppe 2015). The EQ-5D-3L instrument has been validated in a variety of settings, including South Africa and Zimbabwe (Jelsma et al. 2002, 2004), and in patients with diverse conditions, such as arthritis and cancer (Rabin & De Charro 2001). Finally, the instrument yields disability weights or value sets which can be used to calculate quality-adjusted life years (QALYs). This allows the researcher to use preference-based measures to calculate a numerical value for each health state (Dolan 1997).

The measurement tool selected for self-efficacy was the Self-efficacy for Managing Chronic Disease six-item scale (SE-6) (Lorig et al. 2001). The scale was specifically developed in the United States on arthritis patients to test the efficacy of chronic disease education programmes (Brady 1997; Lorig & Holman 1998). The scale was developed from the more comprehensive and arduous Chronic Disease Self-Efficacy Scales that consist of three sections. The sections include the 11-question self-management behaviour section, the 5-question general self-efficacy section and the 17-question self-efficacy section (Lorig et al. 1996). The shorter version of the measurement tool includes the dimensions of emotional functioning of the individual, symptom control, communication with the physician and the role of function in the individual. Participants are required to indicate on a numeric scale from 0 to 10 how confident they are about performing specific activities relating to their chronic disease. Zero indicates that they are ‘not at all confident’ and 10 indicates that they are ‘totally confident’. The scale has been found to be valid and reliable for measuring self-efficacy in chronic conditions in several countries, including individuals living with chronic conditions in France, China, England, Canada, Mexico and Australia (Bélanger et al. 2015; Lorig et al. 1996; Siu et al. 2005); spinal cord injuries in Hong Kong (Pang et al. 2009); DM II in the USA (Fogarty 2007); cancer patients in the UK (Foster et al. 2015); hypertension in United States (Hui Lua et al. 2015), China and South Korea (Hu, Li & Arao 2013) as well as in primary care patients in the United States (Bodenheimer, Lorig & Holman 2002). Validation of the chronic disease self-efficacy scale (SES) has been carried out in Spanish, German, English, Afrikaans, isiXhosa and Zulu, but it has not been translated or validated into Sesotho (Freund et al. 2011; Lacasse et al. 2015; Peltzer et al. 2012; Ritter & Lorig 2014). The instrument was chosen as it had previously been successfully translated into a Bantu language (isiXhosa) and had been used effectively in intervention studies aimed at decreasing pain through improving self-efficacy and exercise (Saw 2015). It is also short and does not take long to administer.

Exercise endurance was assessed with the 6-min walk test. The 6-min walk test is used clinically to determine function in patients with cardiovascular or pulmonary diseases. The 6-min walk test measures the maximum distance that a participant is able to walk in 6 min and is a useful measure because of its similarity to normal activities of daily living. According to Steffen (2002), the test is a more appropriate measure of exercise endurance than maximal exercise capacity (Steffen, Hacker & Mollinger 2002). The test has good test–retest reliability and good construct validity (Enright et al. 2003; Faggiano et al. 1997).

All instruments were either available in English or Sesotho, the local language, translated by linguistics using the standard forward and backward translation procedures, followed by cognitive debriefing to ensure cultural applicability and semantic equivalence.

### Procedure

A pilot study was undertaken with 10 participants to test procedures and check acceptability of the intervention. Nine participants attended baseline measurements and a qualified medical practitioner screened and physically examined the participants to exclude any risk factors. All participants were declared fit to take part in the 3-week pilot intervention programme. After the first week, one participant withdrew because of work commitments, and therefore only eight participants completed the pilot study. The exact testing procedures were followed as described for the actual intervention. The results of the pilot study were excluded in the results of the main study because of the reduced length of the pilot study. The cultural acceptability of the exercises was also considered, and specific adaptations were made based on pilot participant feedback as to cultural acceptability. Participants in the pilot study indicated, during the first exercise session, that they enjoyed ball and dancing activities. The research assistant therefore gave each participant the opportunity during the first session to have a turn to show the type of dance that they enjoyed the most – dancing then served as aerobic exercises during the following sessions. As part of cooling down at the end of the exercise sessions, cultural and traditional dances were encouraged. The participants fully engaged in the sessions by singing traditional songs while performing the dances. As the participants indicated that they enjoyed activities that involved balls, the research assistant after consultation with the researcher included activities utilising balls of different sizes. This resulted in no changes to the protocol.

Participants who had screened positive for MSC a month earlier during the epidemiological survey, who had indicated their willingness and commitment to take part in the intervention, were contacted telephonically to invite them to enrol in the 6-week intervention programme. The researcher (first author) allocated participants to groups as they were recruited into the study. The research assistants who were responsible for administering the outcome measures were blinded as to the allocation. The research assistants were trained by the researcher to ensure that the tests were performed according to the procedures as described in the literature. The experimental group received a date and time for screening and a physical examination by a qualified medical practitioner to assess for risk factors, but no one was excluded on this basis. Once all participants had been cleared for participation, they received a calendar indicating the time and dates for the intervention.

All participants received reminder text messages of the dates for the baseline measures, and in cases where this was not possible, participants were contacted telephonically. An information leaflet containing information describing the details of the study was handed to both groups of participants in their language choice – English or Sesotho. Baseline and follow-up measures were obtained at the PHC clinic. The control group, who were continuing with usual care, were requested to return in 6 weeks for follow-up measures.

At the start of the first session of the intervention, participants were given information to explain why exercise is important, and how to effectively make use of the workbook during the structured education classes as well as at home. The aim of the workbook was to facilitate the development of self-management, decision-making skills, problem-solving skills, and obtaining and utilising resources to assist with managing the chronic disease (Lorig & Holman 2003). The workbook included sections on goal setting, problem-solving tasks and exercise diaries to facilitate skills acquisition. While the control group went about their daily lives as usual, the experimental group attended a weekly educational programme, discussion group and an exercise class lasting 2 h, for 6 weeks at the community hall. In the adapted and developed workbook ‘Balanced lifestyle’, educational topics were specific to the population identified in the survey, which included middle-aged women living with hypertension, diabetes mellitus type II, obesity and MSC. The educational programme aimed at providing information on what each chronic condition was, type and method of exercise deemed appropriate to perform at home, management of common symptoms of MSC, stress management and, finally, healthy food options, including portion sizes according to food-based dietary guidelines with a focus on weight loss. The last educational session included an action plan to continue as a successful self-manager.

The American College of Sports Medicine Guidelines were used to develop the exercise programme, which included aerobic, strengthening and stretching exercises (American College of Rheumatology 2000; Baker & McAlindon 2000; Morey 1999; Van Baar et al. 1999). The researcher chose specific exercises, taking into account the safety needs of an older population and limited availability of equipment. A decision was taken that the duration of the supervised exercise programme would be started at 45 min during the first week and would be increased every week until 60 min of exercise was achieved during the third week, and this would then be maintained for the duration of the programme (American College of Sports Medicine 2010; McDermott & Mernitz 2006). The last 15 min would include physical games for the enjoyment of the participants (Crutzen, Van ’T Riet & Shor 2016). The first author emphasised that the aerobic exercises should be performed according to the Borg Scale level of ‘somewhat hard’ to ensure that participants were exerting themselves at the correct intensity. The level of exertion ‘somewhat hard’ has been found to reflect a 60% effort of maximum heart rate (Borg, Puoane et al. 2012). Appropriate illustrations were used to indicate to participants what was meant by ‘somewhat hard’. The exercise programme was led by a trained research assistant fluent in Sesotho.

Both the control group and the experimental group received clear instructions to adhere to their usual medical treatment. The end-point of data collection was after 6 weeks of the intervention programme. All participants were contacted telephonically to remind them of the date and time for the last measurements to be taken.

As an ethical consideration, the workbook was offered to the control group at the end-point of data collection. Arrangements were made with the community service physiotherapists and the physiotherapy students on the community block to continue with the structured programme at the community clinic on a weekly basis, if the results were shown to be beneficial.

No remuneration was received by participants, other than reimbursement for their travel costs to attend the exercise sessions, as well as the baseline measurements and end-point measurements.

### Data management and statistical analysis

Data were collected in hard copy and then captured by the first author on an Excel spreadsheet and a second person verified the captured data by capturing the data on a separate Excel spreadsheet to ensure accuracy before statistical analyses. Analysis was performed jointly by a statistician, the researcher and one of the supervisors, who, by the nature of the data, were aware of group membership. The York tariff was used to calculate the Index Score (Dolan 1997). The self-efficacy scores were summed and presented out of a maximum of 60 points. The results of those in the control group who did not attend for the second assessment at 6 weeks were excluded from analysis. Descriptive statistics were used to describe the demographic characteristics of the participants. Between-group design requires between four and eight times more subjects than a within group design to reach statistical power (Bellemare, Bissonnette & Kröger 2014). As the study was underpowered and there were discrepancies at baseline in some variables, within-group and between-group analysis was performed throughout. In variables in which the baseline scores differed significantly, the *t*-test or sign test was used to compare the means of the differences over time. The paired *t*-test was used for normally distributed, numeric data and the Wilcoxon signed rank or sign test for the ordinal, non-normal data. Statistica (version 7) (StatSoft, Tulsa, Oklahoma, USA) was used for data analysis. Cohen’s *d* was calculated to determine the effect size, either using the *Z* statistic yielded by the Mann–Whitney U or sign tests or the *t* statistic from the *t*-test results using the Lenhart and Lenhard (2016) online calculator. The 95% confidence intervals (CIs) of *d* were calculated using the formula proposed by Lee (2016).

### Ethical consideration

Ethical approval to perform the study was obtained after a blind review from the Human Research Ethics committee of the University of Cape Town (HREC 605/2013) and the Ethics Committee of the University of the Free State (ECUFS 185/2013). The trial was registered as a clinical trial at the Pan African Clinical Trial registry (PACTR201511000689333).

## Results

**FIGURE 1:**
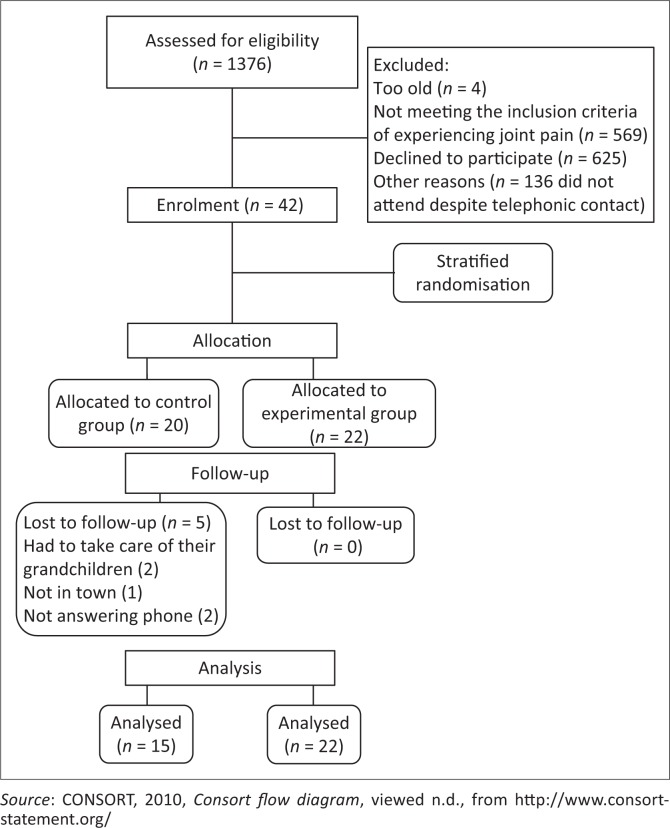
Flow chart of recruitment and participation.

During the survey mentioned above, 1376 patients were assessed, of whom 573 did not have MSC and were not eligible for the intervention study. Of those with MSC, 761 did not wish to commit to a 6-week, weekly intervention. Subsequently, 42 participants were randomly assigned to the two groups, 20 to the control and 22 to the experimental group. Five members of the control group were then lost to follow-up (Figure 1).

The mean age of the participants was 54.5 (SD = 6.8 year, range 40–64 years), and there was no significant difference in age between the two groups. As can be seen in Tables 1 and 2, there were no significant differences in demographic profiles of the groups apart from the larger percentage of unemployed participants and fewer being on pension in the experimental group.

**TABLE 1:** Comparison of the demographic characteristics of the two groups (*n* = 42).

Variable	Categories	Control (*n* = 20) *n* (%)	Experimental (*n* = 22) *n* (%)	*p*
Language	Sesotho	15 (75)	11 (50)	0.285
	IsiXhosa	2 (20)	6 (27)	
	Setswana	3 (15)	5 (23)	
Race	African	20 (100)	22 (100)	Not applicable
Marital status	Married	7 (35)	7 (32)	0.671
	Never married	8 (40)	7 (32)	
	Widowed	4 (20)	6 (27)	
	Separated or divorced	0 (0)	2 (9)	
House ownership	Own	17 (85)	18 (81)	1.000
	Friend or family	3 (15)	4 (18)	
Type of dwelling	Brick	15 (75)	19 (86)	0.445
	Informal	5 (25)	3 (14)	
Number of residents per household	1	1 (5)	2 (9)	0.866
	2	3 (15)	3 (14)	
	3	7 (35)	5 (23)	
	4	2 (10)	4 (18)	
	5	6 (30)	8 (36)	
	6	1 (5)	0 (0)	

**TABLE 2:** Comparison of the education level and employment status of the two groups (*n* = 42).

Variable	Categories	Control (*n* = 20) *n* (%)	Experimental (*n* = 22) *n* (%)	*p*
Literacy	Read or write	20 (100)	22 (100)	1.000
Education	Primary	8 (40)	8 (36)	1.000
	Secondary	12 (60)	14 (64)	
Current employment status	Unemployed	14 (70)	19 (8)	0.003*
	Pensioner	6 (30)	0 (0)	
	Worker – part-time	0 (0)	3 (14)	
Reason for unemployment (*n* = 14 and *n* = 19)	Cannot find work	7 (50)	10 (53)	0.971
	Health problems	6 (43)	8 (42)	
	Family care	1 (7)	1 (5)	
Receive grant benefits	No	12 (60)	16 (73)	0.515
	Yes	8 (40)	6 (27)	
Type of grant benefits (*n* = 8 and *n* = 6)	Disability	1 (13)	4 (67)	0.091
	Pension	7 (88)	2 (33)	

* , values of *p* < 0.05 are viewed as significant.

There was also no significant difference between the health variables of the control group versus the experimental group at baseline and at 6 weeks. The mean attendance rate at classes of the participants in the experimental group was 87.9%.

**FIGURE 2:**
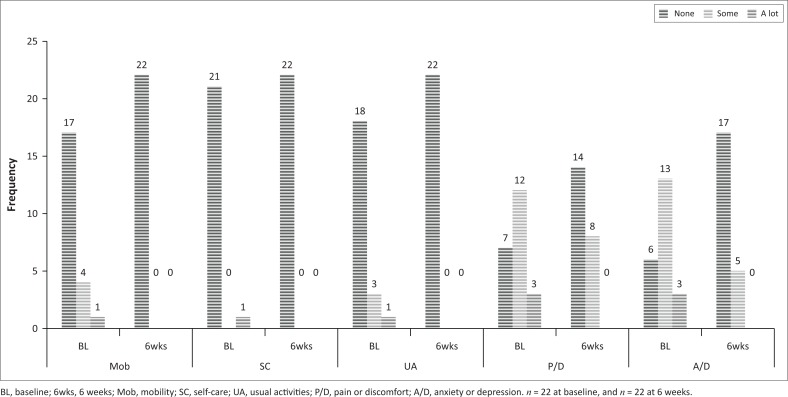
Baseline and 6 weeks frequency of responses to the five dimensions of the EQ-5D-3L for the experimental group.

**FIGURE 3:**
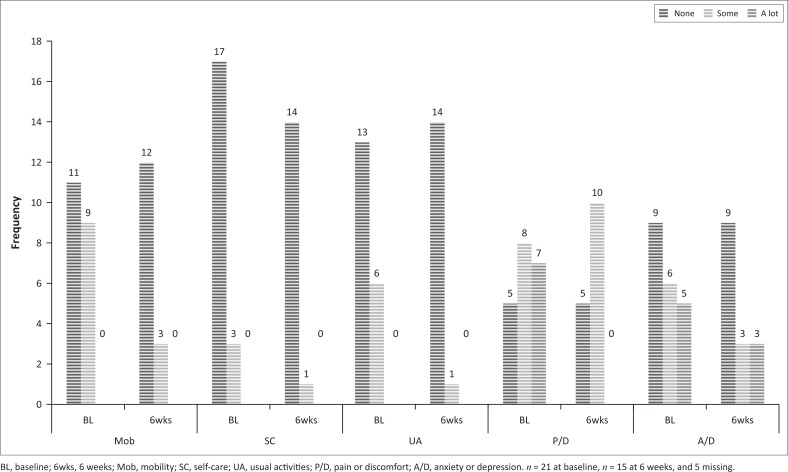
Baseline and 6 weeks frequency of responses to the five dimensions of the EQ-5D-3L for the control group.

As can be seen in Figures 2 and 3, there was a decrease in the relative numbers of participants reporting problems in both groups in the five domains from baseline to the 6-week follow-up. The index and the VAS scores were not normally distributed (Shapiro–Wilks *p* < 0.05), and non-parametric statistics were used for these data. As there were no comparative measurements for the members of the control group who did not return at 6 weeks, their results were also excluded from baseline.

The summary statistics of the VAS and index scores are presented in Table 3. The median EQ-5D-3L index scores in both the control and experimental group increased from baseline to 6 weeks, but this was only significant in the participants in the intervention programme (*p* = 0.010). The experimental group not only improved more (from 0.7 to 1.0) but also showed a significant between-group difference at 6 weeks (*p* = 0.019). There were no significant differences in rank ordering in any of the variables between groups at baseline and at 6 weeks. However, the ranking of mobility and the EQ-5D-3L index had significantly improved in the experimental group at 6 weeks (Table 4). The effect size in both cases was 0.4, indicating a small effect size.

**TABLE 3:** Between-group comparison of rank ordering of health-related quality of life measures at baseline and at 6 weeks.

Variable	Experimental (*n* = 22) Median (IQR)	Control (*n* = 15) Median (IQR)	Adjusted *Z* (Mann–Whitney *U*)	*p*	Effect size Cohen’s *d*
Mobility baseline	1 (1–1)	1 (1–2)	−1.35	0.178	0.46 (−0.45 to 1.95)
Mobility 6 weeks	1 (1–1)	1 (1–1)	−2.13	0.034	0.75 (−0.45 to 1.95)
Self-care baseline	1 (1–1)	1 (1–1)	−0.20	0.844	0.07 (−1.09 to 1.23)
Self-care 6 weeks	1 (1–1)	1 (1–1)	−1.16	0.248	0.39 (−0.78 to 1.56)
Usual activities baseline	1 (1–1)	1 (1–2)	−0.61	0.544	0.20 (−0.97 to 1.37)
Usual activities 6 weeks	1 (1–1)	1 (1–1)	−1.16	0.248	0.39 (−0.78 to 1.56)
Pain or discomfort baseline	2 (1–2)	2 (1.5–3)	−1.54	0.123	−0.52 (−1.70 to 0.66)
Pain or discomfort 6 weeks	1 (1–2)	2 (1–2)	−1.77	0.077	−0.61 (−1.80 to 0.58)
Anxiety or depression baseline	2 (1–2)	2 (1–2.5)	0.39	0.700	0.13 (−1.03 to 1.29)
Anxiety or depression 6 weeks	1 (1–1)	1 (1–1)	−1.37	0.171	0.46 (−0.72 to 1.64)
EQ-5D-3L index score baseline	0.7 (0.4–0.8)	0.7 (0.1–0.7)	0.95	0.341	0.32 (−0.85 to 1.49)
EQ-5D-3L index score 6 weeks	1.0 (0.8–1.0)	0.7 (0.7–1.0)	2.34	0.019	0.83 (−0.38 to 2.04)
VAS baseline	60 (50–70)	50 (50–50)	1.92	0.055	0.31 (−0.86 to 1.48)
VAS 6 weeks	70 (50–80)	60 (50–80)	1.10	0.270	0.18 (−0.99 to 1.35)

VAS, Visual Analogue Scale; IQR, interquartile range.

**TABLE 4:** Within-group comparison of measures at baseline and at 6 weeks.

EQ-5D-3L item	Group	Non-ties‡	Improvement (%)	Sign test *Z* and *p*-value§	Effect size Cohen’s *d* (95% CI)
Mobility	Control†	4	100	*Z* = 1.5 *p* = 0.134	0.5 (−0.66 – 1.70)
	Experimental	9	100	*Z* = 2.7 *p* = 0.008*	1.03 (−0.21 – 2.27)
Self-care	Control†	2	50	*Z* = -0.7 *p* = 0.480	0.24 (−0.93 – 1.41)
	Experimental	3	67	*Z* = 0.0 *p* = 1.000	0 (−1.16 – 1.16)
Usual activities	Control†	3	100	*Z* = 1.2 *p* = 0.248	0.45 (−0.73 – 1.63)
	Experimental	7	100	*Z* = 2.3 *p* = 0.023	0.84 (−0.37 – 2.05)
Pain or discomfort	Control†	12	75	*Z* = 1.4 *p* = 0.149	0.49 (−0.69 – 1.67)
	Experimental	24	79	*Z* = 2.7 *p* = 0.008	1.03 (−0.21 – 2.27)
Anxiety or depression	Control†	9	67	*Z* = 0.7 *p* = 0.505	0.24 (−0.93 – 1.41)
	Experimental	24	79	*p* = 0.008 *Z* = 2.7	1.03 (−0.21 – 2.27)
EQ-5D-3L index score	Control†	15	27	*Z* = 1.5 *p* = 0.121	0.52 (−0.66 – 1.70)
	Experimental	33	21	*Z* = 3.1 *p* = 0.002	1.23 (−0.03 – 2.49)
VAS	Control†	13	31	*Z* = 1.1 *p* = 0.267	0.09 (−1.07 – 1.25)
	Experimental	33	42	*Z* = 0.7 *p* = 0.486	0.24 (−0.93 – 1.41)

VAS, Visual Analogue Scale.

† , only the responses of those who completed both assessments are included.

‡ , the sign test calculate the *Z* statistic calculated based on the non-tied responses, that is, responses which differ from the first to second reading; therefore, the number of non-ties is included.

§ , a positive *Z* score indicates improvement and a decrease in the level of problems reported.

* , values of *p* < 0.05 are viewed as significant.

The median EQ-5D-3L index scores in both the control and experimental group increased from baseline to 6 weeks, but this improvement was only significant for the participants in the intervention programme (*p* = 0.010).

The experimental group showed a significant within-group improvement (i.e. from baseline to 6 weeks) in all of the individual EQ-5D-3L variables apart from self-care and on the VAS. No significant within-group improvements were observed in the control group (Table 4). The effect size over time was large in the mobility (0.9) and usual activities (0.9) domains and medium in pain or discomfort (0.5), anxiety and depression (0.5) domains and the index score (0.5). As the number of non-ties was small in the control group, the effect sizes were not meaningful in most cases.

**FIGURE 4:**
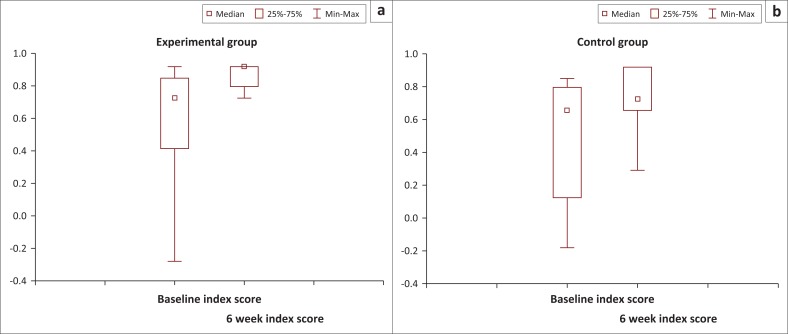
Boxplots of within-group differences of EQ-5D-3L index scores from baseline to 6 weeks. (a), experimental group, *n* = 22, and (b) control group, *n* = 15.

This improvement in the experimental group was further demonstrated by the significant within-group increase in the index scores (Figure 4). The VAS showed no within-group improvement, with the median score of both groups improving 10 points.

The SES showed good internal consistency at baseline with Cronbach’s alpha (α) = 0.887 and excellent internal consistency at 6 weeks after the intervention (α = 0.904). The scores, which are numerical, were normally distributed (Shapiro–Wilks *p* > 0.05) and parametric statistics were used. The control group’s score decreased, or remained the same in every component, apart from confidence in performing tasks. In contrast, the experimental groups’ perceived confidence in every component increased. The difference in fatigue at 6 weeks approached significance (*p* = 0.053) with a medium effect size, compared to small effect sizes for the other items.

There were no within-group differences detected in the control group, but the 6-week scores on the fatigue and discomfort dimensions were significantly greater in the experimental group (*p* = 0.024 and *p* = 0.022, respectively, with medium effect sizes) with a trend towards improvement in managing symptoms (*p* = 0.076, small effect size) (Table 5). Four of the items in the control group showed a negative effect size, that is, the scores decreased from baseline to 6 weeks (Table 6).

**TABLE 5:** Between-group differences from baseline to 6 weeks in the self-efficacy scale.

Item	Mean	Standard deviation	Mean	Standard deviation	*t*	*df*	*p*	Effect size Cohen’s *d*
Fatigue Baseline	5.7	2.71	7.1	3.20	−1.45	40	0.155	0.49 (−0.69 – 1.67)
Fatigue 6 weeks	7.5	2.09	5.9	2.52	2.00	35	0.053	0.67 (−0.52 – 1.86)
Discomfort Baseline	5.6	2.52	6.3	2.98	−0.85	39	0.402	0.29 (−0.88 – 1.46)
Discomfort 6 weeks	7.1	2.18	5.9	2.66	1.45	35	0.156	0.49 (−0.69 – 1.67)
Emotional Baseline	5.9	2.65	7.0	2.82	−1.23	40	0.225	0.41 (−0.69 – 1.67)
Emotional 6 weeks	7.0	2.50	5.9	2.58	1.21	35	0.236	0.41 (−0.69 – 1.67)
Symptoms Baseline	5.0	3.13	6.1	2.95	−1.12	40	0.271	0.35 (−0.82 – 1.52)
Symptoms 6 weeks	6.5	2.61	6.2	2.62	0.34	35	0.734	0.11 (−1.05 – 1.27)
Tasks Baseline	6.4	2.58	7.1	2.38	−0.90	40	0.374	0.30 (−0.87 – 1.47)
Task 6 weeks	6.6	2.11	7.3	2.55	−0.88	35	0.385	0.30 (−0.87 – 1.47)
Do things Baseline	5.9	2.55	7.2	2.30	−1.71	40	0.095	0.57 (−0.62 – 1.76)
Do things 6 weeks	6.09	2.71	5.8	2.83	0.31	35	0.755	0.10 (−1.06 – 1.26)

**TABLE 6:** Within-group differences in self-efficacy items.

Item	Group	Time	Mean	Standard deviation	Difference (SD)	*p*-value and *t*-value	Effect size
Fatigue	Control	Baseline	7.0	3.3	−1.1 (3.5)	Baseline: *p* = 0.26 *t* = −1.17	−0.30 (−1.47 – 0.87)
		After 6 weeks	5.9	2.5			
	Experimental	Baseline	5.7	2.7	1.7 (3.3)	Baseline: *p* = 0.024 *t* = 2.44	0.52 (−0.66 – 1.70)
		After 6 weeks	7.5	2.1			
Discomfort	Control	Baseline	6.3	3.0	−0.4 (2.3)	Baseline: *p* = 0.51 *t* = −0.68	−0.17 (−1.34 – 1.00)
		After 6 weeks	5.9	2.7			
	Experimental	Baseline	5.6	2.5	1.5 (2.7)	Baseline: *p* = 0.022 *t* = 2.49	0.54 (−0.64 – 1.72)
		After 6 weeks	7.0	2.2			
Emotional	Control	Baseline	6.7	2.9	−0.8 (2.6)	Baseline: *p* =0.26 *t* = −1.18	−0.30 (−1.47 – 0.87)
		After 6 weeks	5.9	2.6			
	Experimental	Baseline	5.9	2.7	1.0 (3.2)	Baseline: *p* = 0.144 *t* = 1.52	0.32 (−0.85 – 1.49)
		After 6 weeks	7.0	2.5			
Symptoms	Control	Baseline	6.2	2.8	0.0 (2.5)	Baseline: *p* =1.00 *t* = 0.00	0.00 (−1.16 – 1.16)
		After 6 weeks	6.2	2.6			
	Experimental	Baseline	5.0	3.1	1.86 (3.8)	Baseline: *p* = 0.077 *t* = 1.86	0.40 (−1.16 – 1.16)
		After 6 weeks	6.5	2.6			
Tasks	Control	Baseline	7.1	2.5	0.1 (3.3)	Baseline: *p* = 0.88 *t* = 0.15	0.04 (−1.12 – 1.20)
		After 6 weeks	7.3	2.5			
	Experimental	Baseline	6.4	2.6	0.2 (2.8)	Baseline: *p* = 0.76 *t* = 0.31	0.07 (−1.09 – 1.23)
		After 6 weeks	6.6	2.1			
Do things	Control	Baseline	7.3	2.3	−1.5 (3.8)	Baseline: *p* =0.16 *t* = −1.47	−0.38 (−1.55 – 0.79)
		After 6 weeks	5.8	2.8			
	Experimental	Baseline	5.9	2.6	0.2 (3.3)	Baseline: *p* = 0.750 *t* = 0.32	0.07 (−1.09 – 1.23)
		After 6 weeks	6.1	2.7			

Experimental group, *n* = 21, and control group, *n* =14.

SD, standard deviation.

The overall score of the SE scale for the control group decreased by 3.6, whereas the score of the experimental group increased by 6.45. Neither change was significant, but the mean increase in the overall score of the experimental group shows a trend towards improvement (*p* = 0.055).

The 6-min walk test results are numerical, but as they were not normally distributed (Shapiro–Wilk *p* < 0.05) non-parametric tests were used (Table 7 and Table 8). The experimental group performed significantly better at 6 weeks on the 6-min walk test (*p* = 0.009) than the control group. As the 6-min walk item was significantly different between groups, or approached significance at baseline, the change in score within groups was also calculated. The within-group change from baseline to 6 weeks was only significant in the experimental group (*p* = 0.020).

**TABLE 7:** Between-group comparisons for the 6-min walk test.

Item	Experimental (*n* = 22) Median (IQR)	Control (*n* = 15) Median (IQR)	Adjusted *Z*	*p*-value	Effect size Cohen’s *d*
6-min walk test – baseline (m)	552 (500–578)	445 (420–565)	2.22	0.027	0.78 (−0.43 – 1.99)
6 min walk test – 6 weeks	605 (545–620)	550 (522–595)	2.59	0.009	0.94 (−0.28 – 2.16)

IQR, interquartile range.

**TABLE 8:** Within-group changes for 6-min walk test.

Item	Group	Non-ties	Improvement (%)	*Z* and *p*-value	Effect size Cohen’s
6-min walk test	Control	15	66.7%	*Z* = 103 *p* = 0.302	0.102 (−1.06 – 1.27)
	Experimental	22	77.3%	*Z* = 2.35 *p* = 0.020	0.866 (−0.35 – 2.08)

There were no adverse events in the experimental group. Informal feedback received from the group indicated that there was general satisfaction with the content of the programme. The participants enjoyed the dancing and suggested that more dancing should be added to the programme. All participants requested that the researcher continue with the programme in the future. No negative feedback was received.

## Discussion

The results of the study were encouraging although underpowered. Although not conclusive, the results indicate that the participants appeared to benefit from the intervention, particularly with regard to pain, fatigue and mood.

The recruitment phase was somewhat disappointing, and many of the eligible potential participants did not respond to the recruitment invitation. Although the sample was similar in most respects to the population, the larger number of older and unemployed participants in the experimental sample could indicate that those who were younger and employed were unable to make the time available to attend. As the sample was similar to the population in most respects, the results may be generalised with caution to similar populations, that is, middle-aged women attending PHC who had MSC.

As reported in many studies of community dwelling participants, the most commonly reported problems on the EQ-5D-3L were in the anxiety or depression, pain or discomfort, mobility and performing usual activities domains (Arnold et al. 2004; Jelsma et al. 2004, 2007). The high number of participants reporting pain was as per the inclusion criteria, as all had painful musculoskeletal disorders, but the majority in both groups also reported problems with anxiety or depression (72% across both groups). These figures are higher than the anxiety or depression reported by isiXhosa-speaking individuals living with a disability in urban (50.8%) and rural populations (11.3%) (Jelsma et al. 2007), while slightly lower than the 63.5% in the socially and culturally diverse suburb of Woodstock in Cape Town (Jelsma & Ferguson 2004). The reason for the differences could be because of the socio-economic circumstances of the specific community.

No information pertaining to scores for the 6-min walk test in a similar population could be found. However, the minimal clinically important difference for the 6-min walk test is an increase of 54 m in patients living with chronic obstructive pulmonary disease (Rasekaba et al. 2009). Both the control and the experimental groups improved by more than the minimal clinically important difference. The control group improved by 62.5 m, and the experimental group by 59.4 m. The larger improvement in the control group could be attributed to the testing effect or the demand effect that takes place during research (Cohen et al. 2013). It has been reported by various studies that individuals living with the same amount of pain, but with different levels of self-efficacy, experience different levels of disability. Therefore, the higher the self-efficacy levels, the lower the reported disability (Greene et al. 2006; Marks, Allegrante & Lorig et al. 2005; Wideman & Sullivan 2011; Woby et al. 2007). A study conducted by Oliver and Cronan (2005) clearly indicated that women living with fibromyalgia displaying high levels of self-efficacy showed a decrease in their symptoms and an increase in their physical activity, compared to their counterparts with lower levels of self-efficacy (Oliver & Cronan 2005).

There was evidence that the intervention resulted in improvement in the EQ-5D-3L domains of pain or discomfort, anxiety or depression and the SES items of perceived ability to manage fatigue and discomfort. In addition, there was a trend towards improved mobility in the EQ-5D-3L and in the overall SES scores in the experimental group. The importance of including culturally acceptable and enjoyable items in the programme (Crutzen et al. 2016), such as dancing, was highlighted in the programme which improved the mood of the participants. It is possible that the SES items improved through the education sessions and the interaction with their peers. Despite a lack of improvement in the impairment and functional measures, the participants perceived their HRQoL as having improved considerably, as measured by the EQ-5D-3L index score. The inadequate increase in exercise dosage could be the reason that no impact was noted (Sattelmair et al. 2011) in the distance walked in 6 min. The lack of effect on impairment and health condition needs to be addressed with more frequent sessions over a longer period of time (e.g. 6 months) and a greater dosage (twice a week for a longer period of time) possibly resulting in an improvement in these parameters. At least 150 min of moderate-intensity physical activity or 60 min of vigorous intensity aerobic activity per week (Haskell et al. 2007) is necessary to bring about health benefits and fitness for an individual. Lastly, improving physical functioning will optimise the quality of life, overall well-being, and may possibly improve overall long-term outcomes (American College of Sports Medicine 1998; King & King 2010; Painter 2008; Physical Activity Guidelines Advisory Committee 2008; Saltin et al. 1968). It has been well established in the literature that exercise can improve emotional health, depression, fatigue and self-efficacy thereby improving chronic disease management (Beniamini et al. 1997; Dilorenzo et al. 1999; McAuley & David 1995; Ewart et al. 1986; Rejeski et al. 1998; Singh, Clements & Fiatarone 1997). In addition, moving forward an inter-professional intervention may be utlised, where the dietary sessions are managed by a dietician and the self-management skills by a psychologist. It would also be valuable to recruit local community health workers as peer trainers, who have a good understanding of the local culture.

In both groups, the utility score improved significantly from baseline, but the improvement in the median was 0.2 in the experimental group, compared to 0.1 in the control group. A possible explanation for the improvement in both groups is the testing effect or the demand effect that takes place during research (Cohen et al. 2013), and it is highly unlikely that the improvement was because of standard care, as standard care at the clinic does not address the joint pain of the women. The experimental group gained a median of 0.19 points on the index score, which was significantly more than that of the 0.07 in the control group. An improvement of 0.21 point on the index score (utility score × years lived in that state) implies that the effect on the experimental group of the intervention lasted for one year. To put this differential QALY gain into perspective, the National Institutes of Health Care and Excellence of the UK (NICE), which uses the EQ-5D-3L index to calculate the cost-utility of different interventions, suggests that £20 000–£30 000 is a reasonable amount to pay per QALY (Dillon 2015; McCabe, Claxton & Culyer 2008). The gain of the experimental group would justify the expenditure of approximately £2500 or R50 000 at current exchange rates. Although the threshold cost of a QALY gain in a high-income country, such as the UK, would be far higher than in South Africa, the gain in HRQoL in the experimental group is clearly considerable. Further, cost-utility analysis is recommended to determine what the actual cost of the intervention offered was, but this was beyond the scope of this study.

As in previous studies, the EQ-5D-3L and the SES performed well with regard to the domain scores. In contrast, the EQ-5D VAS did not demonstrate greater improvement in either group from baseline to 6 weeks, which was unexpected. The median scores were low, 50–60, even when compared to those of similar groups in South Africa. The mean EQ-5D-3L score was 60.4 for individuals living with HIV, in the high-density suburb of Khayelitsha, in Cape Town (Hughes et al. 2004); 61.7 in a resource-poor community in the Western Cape, before HAART treatment (Jelsma et al. 2005); 66.1 in isiXhosa-speaking individuals with disabilities in a rural community and 60.3 in an urban community (Jelsma et al. 2007). The lower scores in this study could be attributed to different cultures, and differences in the age groups of study participants as well as the inclusion of male participants in one of the previous studies quoted (Jelsma et al. 2007).

However, the validity of the VAS in this population may be questionable. Forty-two per cent of the respondents scored 50 on the scale, which may indicate a lack of understanding of what the scale represents. In addition, concurrent validity was poor as there was no correlation between the EQ-5D-3L index and the VAS scores. The validity of the isiXhosa version of the VAS of the EQ-5D-3L was established in an urban, under-resourced area (Jelsma et al. 2004; Maart, Jelsma & Amosun 2015), in Cape Town. The poor performance of the EQ-5D-3L VAS suggests there might be a discrepancy between the numeracy levels of populations for which the questionnaire was validated and the population of this study. Poor numeracy and literacy skills are of grave concern in South Africa (Modisaotsile 2012; Spaull 2013), particularly in rural areas, and it would seem that, despite the inclusion criterion of literacy, the participants did not have an adequate level of numeracy to respond appropriately. Additionally, the concepts used in the questionnaires might have been foreign to a population that is naive to research, especially with regard to evaluative questions regarding their health and physical activity levels.

The SES performed better than the EQ-5D-3L and VAS, but there were some aspects that require caution. The internal consistency was high and there was a trend towards correlation with the EQ-5D-3L index score. However, there was some clustering around mid-scores (5 for individual scores and 30 for the total) and full scores (6 for individual and 60 for the total). A possible explanation for this result is that the participants were insufficiently numerate to respond appropriately.

Despite a lack of economic resources, high compliance with attendance (88%) indicates that the intervention was well received and appropriate for this group of women. The feedback received regarding the sessions was consistently positive. The acceptability questionnaire revealed that the participants had all enjoyed the intervention, and apart from including more dancing and games, they would not change the programme and would like it to continue. However, the timing of the intervention (e.g. on weekdays and during the day) may be a factor that could be altered in the future. If classes were offered outside of working hours, it would then allow more participants to attend.

## Limitations

The intervention was based on information gained from several sources and was modified after input from participants in a pilot study. However, more active input from eligible participants in the programme at the planning stage would have been particularly useful, and this is acknowledged as a weakness of the development process. More engagement with the community might have led to further enrichment of the process, and unfortunately, the researcher had no public involvement in the development of the intervention other than the involvement of the 10 women who provided input into the piloting of the programme. The participants who met the inclusion criteria of the study provided input, which resulted in modification of the programme. The pilot study and subsequent changes made ensured the unique local perspective of certain activities in the intervention. More engagement with the community might have led to further enrichment of the process. However, the intervention was piloted on participants who would have met the inclusion criteria of the study and their comments were solicited and resulted in modification of the programme. The pilot study and subsequent changes made did ensure that the unique local perspective towards certain activities in the intervention informed the intervention.

It is acknowledged that there may be room for bias as the first author was involved in allocation to groups (allocation concealment was not performed), in supervising the intervention and in data analysis. However, the allocation by the Excel program was rigorously applied, measurements were taken by blinded assistants and the joint analysis ensured objectivity in statistical analysis.

A fundamental limitation of the study was the small sample size, which may have resulted in a Type II error. The small sample size and non-parametric nature of some of the outcome variables meant that between-group differences were not examined through the use of additional tests such as repeated measures ANOVA to test for time and group interactions. In future studies, it is recommended that the samples would be large enough to support this additional analysis, which requires greater numbers to detect significant differences. The small sample size was because of both a lower than expected recruitment rate and attrition in the control group particularly. This was a pragmatic study and the relatively low uptake is of concern. Because of financial constraints and other demands on time, it is known that individuals from low-income groups are more difficult to recruit successfully for interventions (Anderson 2007; Marcus et al. 2006; Shah et al. 2009). Although the sample was similar in most respects to the population, there was bias in recruitment towards unemployed, older women, and it was clear that younger women in employment were unable to make the time available to attend. A further limitation, despite the inclusion criterion of literacy, was that the participants did not appear to have an adequate level of numeracy to respond appropriately to the questionnaires included in the study. However, even if the intervention demonstrated efficacy under more controlled conditions, this pragmatic trial indicates that the effectiveness may be compromised by poor uptake. This will need to be addressed, not only in future studies but also in service provision. It is acknowledged that there are other methodologies, such as participatory action research, which might yield rich data, and in future studies, it might be useful to use these. However, the RCT is acknowledged as the gold standard for proving the efficacy of interventions (Spieth et al. 2016). It was hoped that the evidence generated by this study would result in the introduction of similar programmes by the health authorities in other PHCs.

## Implications for physiotherapy practice

The positive response to the programme and high compliance with attendance in those who participated as well as the positive way in which the programme was received both indicate that there is a place for group-based interventions incorporating physical activity and health education within the PHC setting within these communities. In addition, the increase in QALYS suggests that the programme may well be cost-effective. The low recruitment rate, which favoured unemployed, older women, might require that the timing of the intervention be examined and late afternoon or weekend classes could perhaps have a greater uptake.

Physiotherapists and possibly other health care professionals should actively participate in the development of appropriate methods of intervening at community level through the medium of PHC clinics. By doing so, they can reduce the negative impact on the functioning and quality of life of those individuals with MSC.

The research study was self-funded and unfortunately not able to continue once the programme was ended. However, information has been used to plan placements for physiotherapy students and has been shared with health authorities, who have been encouraged to make posts available to provide the care for this neglected population.

## Conclusion

This was a pragmatic study, which ultimately included a smaller sample size than planned. In addition, there were concerns about the validity of the outcome measure, which relied on numerical literacy. However, the intervention appears to have promise, and further investigation is warranted. It may thus be useful to regard this as a pilot study for a larger, multi-centre intervention.

It is important that health care professionals as well as policymakers should explore and improve interventions, to empower groups of disadvantaged people who are often neglected by the health system. These interventions should attempt to change the individuals’ health behaviours and reduce existing health inequalities. Establishing a quality PHC physiotherapy service will ensure a comprehensive response to a growing health care burden, especially in resource-poor settings and in the long term may have a potential economic impact on health care in South Africa. There is a definite shift in responsibilities of the day-to-day management of chronic diseases away from the health care professional to the individual living with the chronic disease. Self-management may be the mechanism to bridge the gap between the patient’s needs and the capacity of the present health system services to meet the needs of individuals living with chronic diseases.

## References

[CIT0001] Alford L., 2006, ‘On differences between explanatory and pragmatic clinical trials’, *New Zealand Journal of Physiotherapy* 35, 12–16.

[CIT0002] American College of Rheumatology, 2000, ‘American College of Rheumatology Subcommittee on osteoarthritis guidelines: Recommendations for the medical management of osteoarthritis of the hip and knee’, *Arthritis & Rheumatism* 43, 1905–1915. 11014340 10.1002/1529-0131(200009)43:9<1905::AID-ANR1>3.0.CO;2-P

[CIT0003] American College of Sports Medicine, 1998, ‘ACSM position stand: Exercise and physical activity for older adults’, *Medicine & Science in Sports & Exercise* 30, 992–1008. 9624662

[CIT0004] American College of Sports Medicine, 2010, *ACSM’s guidelines for exercise testing and prescription*, Wolters Kluwer and Lippincott Williams & Wilkins, Baltimore, MD.

[CIT0005] Anderson A.S., 2007, ‘Dietary interventions in low-income women – Issues for UK policy’, *Nutrition Bulletin* 32, 15–20. 10.1111/j.1467-3010.2007.00612.x

[CIT0006] Arnold R., Ranchor A.V., Sanderman R., Kempen G.I.J.M., Ormel J. & Suurmeijer T.P.B.M., 2004, ‘The relative contribution of domains of quality of life to overall quality of life for different chronic diseases’, *Quality of Life Research* 13, 883–896. 10.1023/B:QURE.0000025599.74923.f2 15233502

[CIT0007] Baker K. & Mcalindon T., 2000, ‘Exercise for knee osteoarthritis’, *Current Opinion in Rheumatology* 12, 456–463. 10.1097/00002281-200009000-00020 10990187

[CIT0008] Barnes R.Y., 2016, ‘An investigation into the nature and prevalence of musculoskeletal conditions among women attending a community clinic, and the effectiveness of an intervention programme for these patients’, unpublished doctoral thesis, University of Cape Town, Cape Town, South Africa.

[CIT0009] Barnes R., Jelsma J. & Parker R., 2018, ‘Musculoskeletal conditions within adult midde-aged women, attending a community clinic in a rural area in South Africa: A cross sectional survey’, *Disability and Rehabilitation*, viewed 19 May 2018, from https://protect-za.mimecast.com/s/_DbYCNxKq0iM398oIj9U-J. 10.1080/09638288.2018.1428368 29347849

[CIT0010] Bellemare C., Bissonnette L. & Kröger S., 2014, *Statistical power of within and between-subjects designs in economic experiments*, IZA Discussion Paper No 8583, viewed 19 May 2018, from http://ftp.iza.org/dp8583.pdf.

[CIT0011] Bélanger A., Hudon C., Martin Fortin M., Amirall J., Bouhali T. & Chouinard M.-C., 2015, ‘Validation of a French-language version of the health education impact Questionnaire (heiQ) among chronic disease patients seen in primary care: A cross-sectional study’, *Health and Quality of Life Outcomes* 13, 1–9. 10.1186/s12955-015-0254-0 26306793 PMC4549914

[CIT0012] Beniamini Y., Rubenstein J.J., Zaichkowsky L.D. & Crim M.C., 1997, ‘Effects of high-intensity strength training on quality-of-life parameters in cardiac rehabilitation patients’, *American Journal of Cardiology* 80, 841–846. 10.1016/S0002-9149(97)00533-X 9381995

[CIT0013] Bodenheimer T., Lorig K. & Holman H., 2002, ‘Patient self-management of chronic disease in primary care’, *JAMA* 288, 2469–2475. 10.1001/jama.288.19.2469 12435261

[CIT0014] Brady T.J., 1997, ‘Do common arthritis self-efficacy measures really measure self-efficacy?’, *Arthritis Care & Research* 10(1), 1–8. 9313384 10.1002/art.1790100102

[CIT0015] Brooks P., 2005, ‘Issues with chronic musculoskeletal pain’, *Rheumatology (Oxford)* 44, 831–833. 10.1093/rheumatology/keh648 15840598

[CIT0016] Brooks P.M., 2006, ‘The burden of musculoskeletal disease – A global perspective’, *Clinical Rheumatology* 25, 778–781. 10.1007/s10067-006-0240-3 16609823

[CIT0017] Carmona L., Ballina J., Gabriel R. & LaVon A., 2001, ‘The burden of musculoskeletal diseases in the general population of Spain: Results from a national survey’, *Annals of Rheumatic Diseases* 60, 1040–1045. 10.1136/ard.60.11.1040 PMC1753418 11602475

[CIT0018] Chopra A., 2009, ‘Community rheumatology in India’, *Indian Journal of Rheumatology* 4, 119–126. 10.1016/S0973-3698(10)60192-6

[CIT0019] Cohen L., Manion L., Morrison K., Howell D., Mcmillan J.H. & Schumacher S., 2013, ‘First steps in research’, in Maree K. (ed.), *First steps in research*, Van Schaick Publisher, Pretoria.

[CIT0020] Connelly L.B., Woolf A. & Brooks P., 2006, ‘Cost-effectiveness of interventions for musculoskeletal conditions’, in Jamison D.T., Breman J.G. & Measham A.R. (eds.), *Disease control priorities in developing countries*, 2nd edn., Worldbank, Washington, DC, viewed n.d., from https://www.ncbi.nlm.nih.gov/books/NBK11713/pdf/Bookshelf_NBK11713.pdf

[CIT0021] COPCORD, *Tools*, viewed 10 February 2013, from http://copcord.org/tools.asp

[CIT0022] CONSORT, 2010, *Consort flow diagram*, viewed n.d., from http://www.consort-statement.org/

[CIT0023] Crutzen R., Van ’T Riet J. & Shor C.E., 2016, ‘Enjoyment: A conceptual exploration and overview of experimental evidence in the context of games for health’, *Games for Health Journal: Research, Development, and Clinical Applications* 5, 15–20. 10.1089/g4h.2015.0059 26699455

[CIT0024] Dillon A., 2015, *Carrying NICE over the threshold improving health and social care through evidence-based guidance*, viewed 16 May 2016, https://www.nice.org.uk/news/blog/carrying-nice-over-the-threshold

[CIT0025] Dilorenzo T.M., Bargman E.P., Stucky-Ropp R., Brassington G.S., Frensch P.A. & Lafontaine T., 1999, ‘Long-term effects of aerobic exercise on psychological outcomes’, *Preventive Medicine* 28, 75–85. 10.1006/pmed.1998.0385 9973590

[CIT0026] Dolan P., 1997, ‘Modeling valuations for EuroQol health states’, *Medical Care* 35, 1095–1108. 10.1097/00005650-199711000-00002 9366889

[CIT0027] Edries N., Jelsma J. & Maart S., 2013, ‘The impact of an employee wellness programme in clothing/textile manufacturing companies: A randomised controlled trial’, *BMC Public Health* 13, 1–9. 10.1186/1471-2458-13-25 23311458 PMC3574831

[CIT0028] Enright P.L., Mcburnie M.A., Bittner V., Tracy R.P., Mcnamara R., Arnold A. et al., 2003, ‘The 6-min walk test a quick measure of functional status in elderly adults’, *Chest* 123, 387–398. 10.1378/chest.123.2.387 12576356

[CIT0029] EuroQol Group, 1990, ‘EuroQol – ‘A new facility for the measurement of health-related quality of life’, *Health Policy* 16, 199–208. 10.1016/0168-8510(90)90421-9 10109801

[CIT0030] Ewart C.K., Stewart K.J., Gillilan R.E. & Kelemen M.H., 1986, ‘Self-efficacy mediates strength gains during circuit weight training in men with coronary artery disease’, *Medicine and Science in Sports and Exercise* 18(5), 531–540. 3773670

[CIT0031] Fagard R.H., 2001, ‘Exercise characteristics and the blood pressure response to dynamic physical training’, *Medicne & Science in Sports & Exercise* 33(6Suppl), S484–S492. 10.1097/00005768-200106001-00018 11427774

[CIT0032] Faggiano P., D’Aloia A., Gualeni A., Lavatelli A. & Giordano A., 1997, ‘Assessment of oxygen uptake during the 6-minute walking test in patients with heart failure: Preliminary experience with a portable device’, *American Heart Journal* 134, 203–206. 9313598 10.1016/s0002-8703(97)70125-x

[CIT0033] Fogarty B., 2007, ‘The Effect of a chronic disease self-management program on symptom management of older adults with type 2 diabetes’, *Washington State University* 1–14.

[CIT0034] Foster C., Breckons M., Cotterell P., Barbosa D., Calman L., Corner J. et al., 2015, ‘Cancer survivors’ self-efficacy to self-manage in the year following primary treatment’, *Journal of Cancer Survivorship* 9, 11–19. 10.1007/s11764-014-0384-0 25028218 PMC4341005

[CIT0035] Foster G., Taylor S.J., Eldridge S.E., Ramsay J. & Griffiths C.J., 2007, ‘Self-management education programmes by lay leaders for people with chronic conditions’, *Cochrane Database of Systematic Reviews* 17, 1–49. 10.1002/14651858.CD005108.pub2 17943839

[CIT0036] Freund T., Gensichen J., Szecsenyi J., Goetz K. & Mahler C., 2011, ‘Evaluating self-efficacy for managing chronic disease: Psychometric properties of the six-item Self-Efficacy Scale in Germany’, *Journal of Evaluation in Clinical Practice* 19, 39–43. 10.1111/j.1365-2753.2011.01764.x 21883720

[CIT0037] Gist M.E. & Mitchell T.R., 1992, ‘Self-efficacy: A theoretical analysis of its determinants and malleability’, *Academy of Management Review* 15, 183–211. 10.5465/amr.1992.4279530

[CIT0038] Greene B.L., Haldeman G.F., Kaminski A., Neal K., Lim S.S. & Conn D.L., 2006, ‘Factors affecting physical activity behaviour in urban adults with arthritis who are predominantly African-American and female’, *Journal of the American Physical Therapy Association* 86, 510–519. 16579668

[CIT0039] Greer D.B. & Ostwald S.K., 2015, ‘Improving adherence in African American women with uncontrolled hypertension’, *Journal of Cardiovascular Nursing* 30, 311–318. 10.1097/JCN.0000000000000152 24785136

[CIT0040] Haskell W.L., Lee I.-M., Pate R.R., Powell K.E., Blair S.N., Franklin B.A. et al., 2007, ‘Physical activity and public health: Updated recommendation for adults from the American College of Sports Medicine and the American Heart Association’, *Circulation* 116, 1081–1093. 10.1249/mss.0b013e3180616b27 17671237

[CIT0041] Hu H., Li G. & Arao T., 2013, ‘Validation of a Chinese version of the self-efficacy for managing chronic disease 6-item scale in patients with hypertension in primary care’, *International Scholarly Research Notices: Public Health* 2013, 1–6.

[CIT0042] Hughes J., Jelsma J., Maclean E., Darder M. & Tinise X., 2004, ‘The health-related quality of life of people living with HIV/AIDS’, *Disability and Rehabilitation* 26, 371–376. 10.1080/09638280410001662932 15204489

[CIT0043] Hui Lua A.Y., Hong L., Bong S.H.S., Yeo J.L.S., Tsang M.L.P., Ong K.Z. et al., 2015, ‘A narrative review of the evaluation and selection of instruments which assess self-efficacy amongst patients with essential hypertension’, *Proceedings of Singapore Healthcare* 25(2), 1–7.

[CIT0044] Janssen M.F., Pickard A.S., Golicki D., Gudex C., Niewada M., Scalone L. et al., 2013, ‘Measurement properties of the EQ-5D-5L compared to the EQ-5D-3L across eight patient groups: A multi-country study’, *Quality of Life Research* 22(7), 1717–1727. 23184421 10.1007/s11136-012-0322-4 PMC3764313

[CIT0045] Jelsma J. & Ferguson G., 2004, ‘The determinants of self-reported health-related quality of life in a culturally and socially diverse South African community’, *Bulletin of the World Health Organization* 82, 206–212. 15112009 PMC2585936

[CIT0046] Jelsma J., Maart S., Eide A., Ka’toni M. & Loeb M., 2007, ‘The determinants of health-related quality of life in urban and rural isi-Xhosa-speaking people with disabilities’, *International Journal of Rehabilitation Research* 30, 119–126. 10.1097/MRR.0b013e32813a2e88 17473623

[CIT0047] Jelsma J., Maclean E., Hughes J., Tinise X. & Darder M., 2005, ‘An investigation into the health-related quality of life of individuals living with HIV who are receiving HAART’, *AIDS Care* 17, 579–588. 10.1080/09540120412331319714 16036244

[CIT0048] Jelsma J., Mkoka S., Amosun L. & Nieuwveld J., 2004, ‘The reliability and validity of the Xhosa version of the EQ-5D’, *Disability and Rehabilitation* 26, 103–108. 10.1080/09638280310001629705 14668147

[CIT0049] Jelsma J.M., De Kock P.A., De Weerdt W.H., Mielke J., 2002, ‘The validity of the Shona version of the EQ-5D quality of life measure’, *South African Journal of Physiotherapy* 58, 8–12. 10.4102/sajp.v58i3.215

[CIT0050] Kelley G.A. & Kelley K.S., 2000, ‘progressive resistance exercise and resting blood pressure: A meta-analysis of randomized controlled trials’, *Hypertension* 35, 838–843. 10720604 10.1161/01.hyp.35.3.838

[CIT0051] King A.C. & King D.K., 2010, ‘Physical activity for an aging population’, *Public Health Reviews* 32, 401–426. 10.4102/sajp.v58i3.215

[CIT0052] Kruger-Jakins T., Saw M., Edries N. & Parker R., 2016, ‘The development of an intervention to manage pain in people with late-stage osteoarthritis’, *South African Journal of Physiotherapy* 72(1), 1–7. 10.4102/sajp.v72i1.311 PMC6093135 30135890

[CIT0053] Lacasse A., Bourgault P., Tousignant-Laflamme Y., Courtemanche-Harel R. & Choinière P.M., 2015, ‘Development and validation of the French-Canadian chronic pain self-efficacy scale’, *Pain Research & Management* 20(2), 75–83. 25848845 10.1155/2015/832875 PMC4391442

[CIT0054] Lee D.K., 2016, ‘Alternative to p value: Confidence interval and effect size’, *Korean Journal of Anesthesiology* 69(6), 555–562. 10.4097/kjae.2016.69.6.555 27924194 PMC5133225

[CIT0055] Lee I.M., Shiroma E.J., Lobelo F., Pushka P., Blair S.N. & Katzmarzyk P.T., 2012, ‘Impact of physical inactivity on the world’s major non-communicable diseases’, *Lancet* 380, 219–229. 10.1016/S0140-6736(12)61031-9 22818936 PMC3645500

[CIT0056] Lenhart W. & Lenhard A., 2016, *Calculation of effect sizes*, viewed 14 August 2018, from https://www.psychometrica.de/effect_size.html

[CIT0057] Lewis M., Eskeland G. & Traa-Valerezo X., 2004, ‘Primary health care in practice: Is it effective?’, *Health Policy* 70, 303–325. 10.1016/j.healthpol.2004.04.011 15488997

[CIT0058] Lorig K. & Holman H., 1998, ‘Arthritis self-efficacy scales measure self-efficacy’, *Arthritis Care and Research* 11, 155–157. 10.1002/art.1790110302 9782806

[CIT0059] Lorig K., Stewart A., Ritter P. & Gonzalez V., 1996, ‘Chronic disease self-efficacy scales’, *Outcome Measures for Health Education and Other Health Care Interventions*, 41–45. 10.4135/9781452232966

[CIT0060] Lorig K.R. & Holman H.R., 2003, ‘Self-management education: History, definition, outcomes, and mechanism’, *Annals of Behavioral Medicine* 26, 1–7. 12867348 10.1207/S15324796ABM2601_01

[CIT0061] Lorig K.R., Sobel D.S., Ritter P.L., Laurent D. & Hobbs M., 2001, ‘Effect of a self-management program on patients with chronic disease’, *Effective Clinical Practice*, 256–262. 10.1207/S15324796ABM2601_01 11769298

[CIT0062] Maart S., Jelsma J. & Amosun S., 2015, ‘Disability in under resourced areas in the Western Cape, South Africa: A descriptive analytical study’, PhD, University of Cape Town.

[CIT0063] Marcus B.H., Williams D.M., Dubbert P.M., Sallis J.F., King A.C., Yancey A.K. et al., 2006, ‘Physical activity intervention studies: What we know and what we need to know: A scientific statement from the American Heart Association Council on Nutrition, Physical Activity, and Metabolism (Subcommittee on Physical Activity); Council on Cardiovascular Disease in the Young; and the Interdisciplinary Working Group on Quality of Care and Outcomes Research’, *Circulation* 114, 2739–2752. 17145995 10.1161/CIRCULATIONAHA.106.179683

[CIT0064] Marks R., Allegrante J.P. & Lorig K., 2005, ‘A review and synthesis of research evidence for self-efficacy-enhancing interventions for reducing chronic disability: Implications for health education practice (part I)’, *Health Promotion Practice* 6(1), 37–43. 10.1177/1524839904266790 15574526

[CIT0065] McAuley E. & David R., 1995, ‘Physical activity, aging, and psychological well-being’, *Journal of Ageing and Physical Activity* 3, 67–96.

[CIT0066] Mccabe C., Claxton K. & Culyer A.J., 2008, ‘The NICE cost-effectiveness threshold what it is and what that means’, *Pharmacoeconomics* 26, 733–744. 10.2165/00019053-200826090-00004 18767894

[CIT0067] McCaffrey N., Kaambwa B., Currow D.C. & Ratcliffe J., 2016, ‘Health-related quality of life measured using the EQ-5D-5L: South Australian population norms’, *Health and Quality of Life Outcomes* 14, 133 10.1186/s12955-016-0537-0 27644755 PMC5028927

[CIT0068] Mcdermott A.Y. & Mernitz H., 2006, ‘Exercise and older patients: Prescribing guidelines’, *American Family Physician* 74, 437–44. 16913163

[CIT0069] Minas M., Koukosias N., Zintzaras E., Kostikas K. & Gourgoulianis K.I., 2010, ‘Prevalence of chronic diseases and morbidity in primary health care in central Greece: An epidemiological study’, *BMC Health Services Research* 10, 252 10.1186/1472-6963-10-252 20799979 PMC2939599

[CIT0070] Modisaotsile B.M., 2012, ‘The failing standard of basic education in South Africa’, *AISA Policy Brief* 72, 1–7.

[CIT0071] Morey S.S., 1999, ‘ACSM revises guidelines for exercise to maintain fitness’, *American Family Physician* 59, 473, viewed n.d., from http://www.aafp.org/afp/1999/0115/p473.html 9930133

[CIT0072] Oliver K. & Cronan T.A., 2005, ‘Correlates of physical activity among women with fibromyalgia syndrome’, *Annals of Behavioral Medicine* 29, 44–53. 10.1207/s15324796abm2901_7 15677300

[CIT0073] Painter P., 2008, ‘Exercise in chronic disease: Physiological research needed’, *Exercise and Sport Sciences Reviews* 36, 83–90. 10.1097/JES.0b013e318168edef 18362690

[CIT0074] Pang M.Y.C., Eng J.J., Lin K.H., Tang P.F., Hung C. & Wang Y.H., 2009, ‘Association of depression and pain interference with disease-management self-efficacy in community-dwelling individuals with spinal cord injury’, *Journal of Rehabilitation Medicine* 41, 1068–1073. 10.1097/JES.0b013e318168edef 19894003

[CIT0075] Parker R., Jelsma J. & Stein D.J., 2016, ‘Managing pain in women living with HIV/AIDS: A randomized controlled trial testing the effect of a six-week peer-led exercise and education intervention’, *Journal of Nervous & Mental Disease* 204, 665–672. 10.1097/JES.0b013e318168edef 27002748

[CIT0076] Parker R.E., 2013, ‘Pain in HIV/AIDS: Characteristics, contributing factors and the effects of a six-week peer-led exercise and education intervention’, PhD in Physiotherapy, University of Cape Town.

[CIT0077] Peltzer K., Ramlagan S., Jones D., Weiss S.M., Fomundam H. & Chanetsa L., 2012, ‘Efficacy of a lay health worker led group antiretroviral medication adherence training among non-adherent HIV-positive patients in KwaZulu-Natal, South Africa: Results from a randomized trial’, *Journal of Social Aspects of HIV/AIDS* 9, 218–226. 10.1097/JES.0b013e318168edef 23234350

[CIT0078] Pfleger B., 2007, ‘Burden and control of musculoskeletal conditions in developing countries: A joint WHO/ILAR/BJD meeting report’, *Clinical Rheumatology* 26, 1217–1227. 17522948 10.1007/s10067-007-0645-7

[CIT0079] Physical Activity Guidelines Advisory Committee, 2008, *Physical activity guidelines*, viewed 16 July 2015, from https://health.gov/paguidelines/report/pdf/CommitteeReport.pdf

[CIT0080] Puoane T.R., Tsolekile L., Igumbor E.U. & Fourie J.M., 2012, ‘Experiences in developing and implementing health clubs to reduce hypertension risk among adults in a South African population in transition’, *International Journal of Hypertension* 2012, 913–960. 10.1097/JES.0b013e318168edef PMC3431082 22957212

[CIT0081] Rabin R. & De Charro F., 2001, ‘EQ-SD: A measure of health status from the EuroQol Group’, *Annals of Medicine* 33(5) 337–343. 10.1097/JES.0b013e318168edef 11491192

[CIT0082] Rasekaba T., Lee A.L., Naughton M.T., Williams T.J. & Holland A.E., 2009, ‘The six-minute walk test: A useful metric for the cardiopulmonary patient’, *Journal of Internal Medicine* 39, 495–501. 10.1097/JES.0b013e318168edef 19732197

[CIT0083] Rejeski W.J., Ettinger W.H.J., Martin K. & Morgan T., 1998, ‘Treating disability in knee osteoarthritis with exercise therapy: A central role for self-efficacy and pain’, *Arthritis Care and Research* 11(2), 94–101. 9668732 10.1002/art.1790110205

[CIT0084] Rollman G.B. & Lautenbacher S., 2001, ‘Sex differences in musculoskeletal pain’, *The Clinical Journal of Pain* 17, 20–24. 11289085 10.1097/00002508-200103000-00004

[CIT0085] Ritter P.L. & Lorig K., 2014, ‘The English and Spanish self-efficacy to manage chronic disease scale measures were validated using multiple studies’, *Journal of Clinical Epidemiology*, 2–9. 10.1097/JES.0b013e318168edef 25091546

[CIT0086] Saltin B., Blomqvist G., Mitchell J.H., Johnson R.L.J., Wildenthal K. & Chapman C.B., 1968, ‘Response to exercise after bed rest and after training’, *Circulation* 38(5), VII1–VII78. 5696236

[CIT0087] Sattelmair J., Jeremy Pertman J., Ding E.L., Harold W., Kohl Iii H.W., Haskell W. et al., 2011, ‘Dose response between physical activity and risk of coronary heart disease a meta-analysis’, *Circulation* 124, 789–795. 10.1097/JES.0b013e318168edef 21810663 PMC3158733

[CIT0088] Saw M., 2015, ‘The effects of a six-week physiotherapist-led exercise and education intervention in patients with osteoarthritis, awaiting an arthroplasty in South Africa’, MSc Physiotherapy Dissertation, University of Cape Town. 10.1186/s12891-016-1088-6 PMC4884378 27233479

[CIT0089] Shah L.M., Arora V., King A. & Krishnan J., 2009, ‘The presence of tobacco cessation programs is not sufficient for low-income hospitalized smokers’, *Archives of Internal Medicine* 169, 902–903. 19433704 10.1001/archinternmed.2009.107

[CIT0090] Singh N.A., Clements K.M. & Fiatarone M.A., 1997, ‘A randomized controlled trial of progressive resistance training in depressed elders’, *Journal of Gerontology: Medical Sciences* 52, 27–35. 10.1093/gerona/52A.1.M27 9008666

[CIT0091] Siu A.M.H., Chan C.C.H., Poon P.K.K., Chui D.Y.Y. & Chan S.C.C., 2005, ‘Evaluation of the chronic disease self-management program in a Chinese population’, *Patient Education and Counseling* 65, 42–50. 10.1016/j.pec.2006.04.013 PMC7135159 16872789

[CIT0092] Spaull N., 2013, *South Africa’s education crisis: The quality of education in South Africa 1994–2011*, Centre for Development and Enterprise, Johannesburg.

[CIT0093] Spieth P.M., Kubash A.S., Penzlin A.I., Illigens B.M.W., Barlinn K. & Siepmann T., 2016, ‘Randomized controlled trials – A matter of design’, *Neuropsychiatric Disease and Treatment* 12, 1341–1349. 27354804 10.2147/NDT.S101938 PMC4910682

[CIT0094] Stajkovic A.D. & Luthans F., 2002, *Social cognitive theory and self-efficacy: Implications for motivation theory and practice*, viewed n.d., from https://www.researchgate.net/publication/258995495_Social_cognitive_theory_and_self-efficacy_Implications_for_motivation_theory_and_practice

[CIT0095] Steffen T.M., Hacker T.A. & Mollinger L., 2002, ‘Age and gender related test performance in community-dwelling elderly people: Six-minute walk test, Berg balance scale, timed up & go test, and Gait speeds’, *Physical Therapy* 82, 128–137. 10.1016/j.pec.2006.04.013 11856064

[CIT0096] van Baar M.E., Assendelft W.J.J., Dekker J., Oostendorp R.A.B. & Bijlsma J.W.J., 1999, ‘Effectiveness of exercise therapy in patients with osteoarthritis of the hip or knee’, *Arthritis & Rheumatism* 42(7), 1361–1369. 10403263 10.1002/1529-0131(199907)42:7<1361::AID-ANR9>3.0.CO;2-9

[CIT0097] Van Reenen M. & Oppe M., 2015, *EQ-5D-3L user guide. 5.1*., April 2015, 1–25, viewed n.d., from https://euroqol.org/wp-content/uploads/2016/09/EQ-5D-5L_UserGuide_2015.pdf

[CIT0098] Wideman T.H. & Sullivan M.J.L., 2011, ‘Differential predictors of the long-term levels of pain intensity, work disability, healthcare use, and medication use in a sample of workers’ compensation claimants’, *Pain* 152, 376–383. 10.1016/j.pain.2010.10.044 21147513

[CIT0099] Woby S.R., Raoch N.K., Urmston M. & Watson P.J., 2007, ‘The relation between cognitive factors and levels of pain and disability in chronic low back pain patients presenting for physiotherapy’, *European Journal of Pain* 11(8), 869–877. 10.1016/j.pain.2010.10.044 17360202

[CIT0100] World Health Organization, 2015, *Obesity and overweight*, Fact sheet 311, viewed 23 February 2015, from http://www.who.int/mediacentre/factsheets/fs311/en/

